# Prevalence and Early Prediction of Diabetes Using Machine Learning in North Kashmir: A Case Study of District Bandipora

**DOI:** 10.1155/2022/2789760

**Published:** 2022-10-04

**Authors:** Salliah Shafi Bhat, Venkatesan Selvam, Gufran Ahmad Ansari, Mohd Dilshad Ansari, Md Habibur Rahman

**Affiliations:** ^1^B. S. Abdur Rahman Crescent Institute of Science and Technology, Chennai 48, India; ^2^MIT World Peace University (MIT-WPU), Pune 411 038, India; ^3^Guru Nanak University, Hyderabad501506, TS, India; ^4^Department of Computer Science and Engineering, Faculty of Engineering and Technology, Islamic University, Kushtia 7003, Bangladesh

## Abstract

Diabetes is one of the biggest health problems that affect millions of people across the world. Uncontrolled diabetes can increase the risk of heart attack, cancer, kidney damage, blindness, and other illnesses. Researchers are motivated to create a Machine Learning methodology that can predict diabetes in the future. Exploiting Machine Learning Algorithms (MLA) is essential if healthcare professionals are able to identify diseases more effectively. In order to improve the medical diagnosis of diabetes this research explored and contrasts various MLA that can identify diabetes risk early. The research includes the analysis on real datasets such as a clinical dataset gathered from a doctor in the Indian district of Bandipora in the years April 2021–Feb2022. MLA are currently important in the healthcare sector due to their prediction abilities. Researchers are using MLA to improve disease prediction and reduce cost. In this Paper author developed a methodology using Machine Learning Algorithms for Diabetes Disease Risk Prediction in North Kashmir. Six MLA have been successfully used in the experimental study such as Random Forest (RF), Multi-Layer Perceptron (MLP), Support Vector Machine (SVM), Gradient Boost (GB), Decision Tree (DT), and Logistic Regression (LR). RF is the most accurate classifier with the uppermost accuracy rate of 98 percent followed by MLP (90.99%), SVM (92%), GBC (97%), DT (96%), and LR (69%), respectively, with the balanced data set. Lastly, this study enables us to effectively identify the prevalence and prediction of diabetes.

## 1. Introduction

Diabetes is a chronic metabolic disorder characterised by an increased blood glucose level brought on by insufficient insulin production [1]. According to statistics 285 million people globally had diabetes in 2010. By 2030 it is estimated that there would be 552 million people living worldwide (6.4 percent of adults) [2]. By 2040 diabetes was projected to have developed in one in ten people based on the disease current growth rate [3]. In India the prevalence of diabetes has considerably increased as well current research reveals that out of 100000 people by 2040.124874.7 people will suffer from diabetes [4]. Two out of every five persons have diabetes and one in four have Prediabetes [5]. Overall numerous people are suffering with the serious condition of diabetes. Diabetes is a widespread chronic condition. A challenging task is making early prediction and diagnosis of diabetes. As a result diabetes disease prediction requires an efficient and accurate method. Further due to the nature of the diseases diabetes frequently goes undiagnosed because persons who have diabetes are frequently unaware of their condition or are themselves asymptomatic [6]. The kidneys, heart, nerves, blood arteries, and eyes are just a few of the body systems and organs that suffer severe long-lasting damage from uncontrolled diabetes. Consequently, early disease detection enables patients who are at risk to take preventative processes to slow the disease progression and improve the quality of life. Imbalanced data detection remains one of the major challenges in the field. The primary learning algorithm makes the assumption that classes in the training dataset are generally balanced. Usually learning outcome variables make the assumption that the classes in the dataset are all of identical importance. However, balanced datasets are rare in authentic situations and the underrepresented class typically experiences increased misdiagnosis. A prediction model with a deceptively high accuracy of 95.4% will classify all the majority classes properly while misclassifying all the minority classes. Serious consequences may result if people with diabetes are misclassified. Machine learning can use past outcomes to make smart decisions on present cases that were before unknown. Machine-learning classifiers are utilized in this research for classification. To predict diabetes disease Machine Learning classifier is trained by using a dataset. The classifiers employed in this research include RF, KNN, MLP, SVC, GB, DT, and LR. Diabetes is a hereditary illness that develops while the pancreas does not contain enough insulin. There have been significant improvements in health care services using trimming technology as a result of the technological rapid growth like AI and ML. Machine Learning as well as artificial intelligence to reduce the consequences of diabetes and enhance the standard of patient care [7]. Numerous researchers have developed ML-based techniques for predicting the incidence of diabetes [8]. The aim of this work is to developed ML techniques that use clinical data from a rural area of North Kashmir to forecast the prevalence and prediction of early diabetes. The prediction models classify each occurrence of the input data into one of two conditions: normal (nondiabetic) Prediabetes or diabetes. Key features were first chosen using a data-driven feature selection strategy that combined a statistical and feature reduction techniques in order to develop the prediction method. Moreover, we analysed the effectiveness of the RF, MLP, SVM, GBC, DT, and LR. The main objective of this research is as follows:

- Six Machine Learning Algorithms were used, namely, RF, MLP, GNB, SVC, GB, DT, and LR
- Feature selection is used for the purpose of finding the important variables
- Data balancing is used in order to balance the data for finding the best accuracy
- Proposed a Methodology for Machine Learning techniques for diabetes Risk Prediction

The proposed research is split into the following sections: Section 2 discusses earlier significant work on early prediction based on Machine Learning techniques in Literature Review. We discussed the methodology in Section 3 including data collection, data set description, data pre-processing, feature engineering, and Machine Learning algorithms. In Section 4 we have discussed the model accuracy. We talked about the result and discussion in Section 5 of the paper.  Section 6 concludes and future work highlighting its limitations and offers suggestions for future research that can improve early diabetes prediction.

## 2. Literature Review

The related work section provides a brief explanation of earlier literature studies on Machine Learning based diabetes predictive systems. With aid of MLA, many studies have attempted to address the problem of diabetes prediction. These studies include Support Vector Machine (SVM), DT, NB, and ANN. Early diabetes diagnosis is crucial since it can be made when the disease is still in its early stages [9]. A thorough investigation of machine learning techniques for detecting diabetes. Two crucial data processors, Principal Component Analysis as well as Linear Discriminant Analysis was examined in the study for use with diverse Machine Learning techniques. Experimentation was used to establish the optimal data preprocessing for each algorithm, and parameter changes were made to achieve the best performance. To evaluate how well the algorithms worked in Pima Indian data collection was used. The accuracy that was achieved using 10-fold cross-validation was 77.86 percent across the five algorithms (neural network, SVM, DTM, LR, and Naive Bayes) that were used. For the categorization of diabetes, Qawqzeh et al. recommended an LR model based on the investigation. Moreover, the author used data from 459 patients for testing and model validation and 128 data points for training. 552 people were accurately recognized as nondiabetics using the suggested approach, which had a 92 percent accuracy rate. The developed technique is not contrasted with cutting-edge methods, nevertheless. Using MLA Pethunachiyar presented a system for categorising diabetic mellitus. They used an SVM principally, along with data on diabetes from the UCI Machine Repository, and a number of kernel functions. In comparison to naive Bayes, decision trees, and neural networks we found SVM with a linear function to be more effective. Though there is no state-of-the-art assessment and the variable decision is not explained. MLA was employed by Maniruzzaman et al. [10] to classify and forecast diabetes. For the classification of diabetes, they used four MLA NB, DT, AdaBoost, and RF. For enhanced outcomes they combined three alternative partition protocols with the 20 experiments. People with and without diabetes were surveyed in the US for the National Health and Nutrition Survey and they saw encouraging findings from the suggested technique. Ahuja et al. [11] classified diabetes patients using the PIMA dataset, and conducted a comparison analysis of several MLA, including NB, DT, and MLP. They observed that MLP performed improved than other classifiers. Effective feature engineering and fine-tuning, according to the authors, can boost MLP performance. Moreover, a soft computing technique for diabetes prediction scheme developed by Kumari et al. [12] makes use of an ensemble of three popular supervised MLA. For evaluation, the authors have utilized the PIMA and breast cancer databases. The accuracy of their system exceeds state-of-the-art individual and ensemble techniques by 79 percent were achieved using RF, LR, and NB. Further tested with the PIMA Indian dataset and the BUPA liver difficulties dataset to find individuals with diabetes as well as liver illnesses after balancing and extending the research using the PIMA data the accuracy was 83.57 percent and 86.36 percent. In a variety of studies, a group of investigators obtained data from the National Health and Nutrition Examination Survey in order to make a Machines Learning based scheme to forecast diabetic patients. The suggested approach showed that, for the K10 protocol, the logistic regression feature extraction and random forest classifier had the greatest accuracy of 94.25 percent with 0.95 AUC [13]. On the PIMA Indian dataset and the dataset from Tabriz, Iran, Zaharias, and colleagues employed cost sensitive learning [14]. Moreover, to find the best algorithm for precise diabetes prediction, Faniqul et al. obtained a dataset of 520 instances from Sylhet Diabetes Hospital Bangladesh. They tested various MLA using 10-fold cross validation and obtain that RF performed the best with an accuracy of 97.4%. SVM performed better and was more accurate than other algorithms, according to Kavakiotis et al. study of 3 different algorithms (LR, NB, and SVM) using ten-fold cross-validation [15]. These suggested aggregation methods that choose the classification parameters using the ANN, NB, KNN, J48, simple cart, and filtered classifier. A 77.01 percent accuracy rate was achieved using the suggested methods. Li L. proposed a method for classifying data using SVM, ANN, Nave Bayes, and a weighted-based research. A 77.01 percent accuracy rate was achieved using the suggested methods. Li L. proposed a technique for classifying data using SVM, ANN, Nave Bayes, and a weighted-based research. Kumari et al. recommended an ensemble model of CART, ID3, and C4.5 that had 76.5 percent accuracy [16]. By collecting 520 records and interviewing patients at the Sylhet Diabetes Hospital Islam et al. [17] conducted a study. During the research period the algorithms NB, LR, and RF were applied. Random Forest was depicts to have the best accuracy on their test database after being evaluated using Tenfold Cross Validation and Percentage Split [10, 18–22]. In the end, they have suggested a simple method for the user to determine diabetes by assessing their characteristics. Understanding diabetes is important for biological treatment. The main objective is to forecast Prediabetes using AI and ML [10, 14–27]. In this research work, the author used well-known MLA approaches to examine actual diagnostic medical data based on various risk factors to assess their effectiveness for diabetic probability. Seven MLA were used in this study such as RF, KNN, MLP, SVC, GBC, DT, and LR [24–32]. Various statistical criteria were used to compare the analytical results. Implementation and analysis allowed RF to outperform every classifier with an accuracy of 98%. A comparative study of previous research regarding performance parameters is displayed in Table 1.

## 3. Proposed Methodology

**Figure 1:**
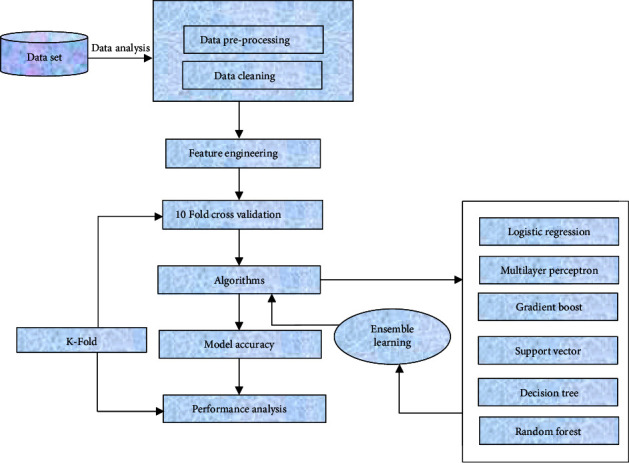
Proposed methodology for machine learning algorithms for diabetes disease risk prediction.

To obtain datasets, feature engineering model construction based on MLA and performance evaluation are described. The working flow of methodology used for this research work is shown in Figure 1. The data analysis file was created using clinical diabetic data. It explains the sequential steps required to construct a realistic framework using machine learning methods based on the ensemble learning.

**Step 1 .**

Data preparation has utilized in data balance, testing, and training.

**Step 2 .**

After that, appropriate evaluations model specifically the tenfold cross-validation and percentage split methods will be employed to examine the efficiency of the models.

**Step 3 .**

The dataset comprising data on patients symptoms will be placed into prediction algorithms such as RF, MLP, SVC, DT, and LR algorithms. The system is then developed for the end users using the best algorithm and the data as the storage.

**Step 4 .**

After choosing the most accurate classification models, ensemble learning methods have finally been implemented.

**Step 5 .**

This algorithm will assist the user in risk prediction using the symptom as data. It includes the methods for implementing MLA to diagnose diseases from data collection to the specific goals.

### 3.1. About Dataset

**Figure 2:**
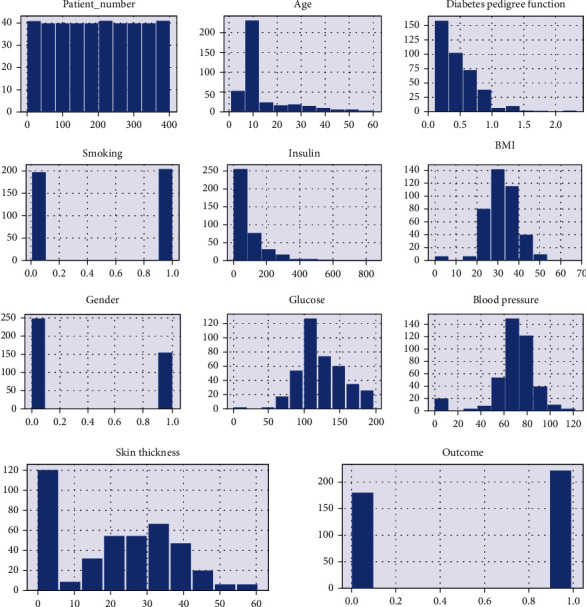
Histogram of each attribute.

**Figure 3:**
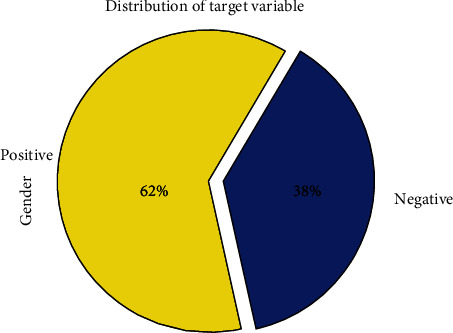
Relation between positive and negative i.e., (diabetic vs. nondiabetic).

We collected the clinical dataset using the snow sampling technique by collaborating with a clinical diabetic professional. The collected dataset has 403 instances each with 11 attributes. The dataset does not contain any personal information such as the names of the person or their personal identification numbers in order to protect their privacy. However, the data is imbalanced. There are multiple techniques through which we can remove in balancing factors so that overall accuracy can be improved. The experimental study's dataset, which was constructed using clinical data in accordance with the endocrinologist's recommendations (diabetes specialists). The chosen characteristics are shown in Figure 2. By arranging up a brief conversation with the patients a group of medical residents was contacted to gather the dataset. The data collection process took eleven months (From April 2021 to Feb 2022). The data set description is shown in Figure 3.

Further Figure 3. Represents the outcome target i.e. diabetes vs. nondiabetic here zero represents nondiabetic and one represents diabetic.

### 3.2. Data Reprocessing

The clinical dataset was used to evaluate and test the suggested method. In the clinical dataset, there are many different types of disorders. The raw data are transformed into a data analysis file format for cleaning and extraction of features. The methodology presented in this article defines diabetic medicine. The patient is different from the optimum healthy patient.

#### 3.2.1. Data Cleaning

Unprocessed information was acquired from data. Due to this many techniques have been used to clean the data such as deleting duplicates and irrelevant data.

#### 3.2.2. Data Balancing

**Figure 4:**
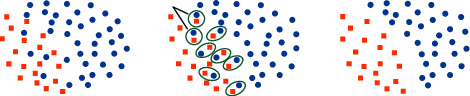
Tomek Links: under sampling.

**Figure 5:**
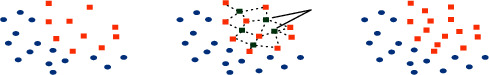
Synthetic minority oversampling.

Classifications that are not equally balanced make prediction modelling more difficult. MLA for classification usually starts with an equal set of examples for each class they are attempting to understand. To provide better precise and accurate findings, incorrect data are handled and removed in this phase. Missing values can be found in this data set such as Patient-NumberAge, Diabetes Pedigree Function, Smoking, BMI, Insulin, Skin Thickness, Blood Pressure, Gender Glucose, and result. These parameters are assigned with blank values since they cannot have null values. By scaling the data set we balanced all values. This research has led to a major evolution in resampling techniques. For instance, by removing records from each group most of the classifier can be combined, and under sampling can be performed. Figures 4 and 5 show the outcomes of applying under sampling and oversampling methodologies, respectively.

**Figure 6:**
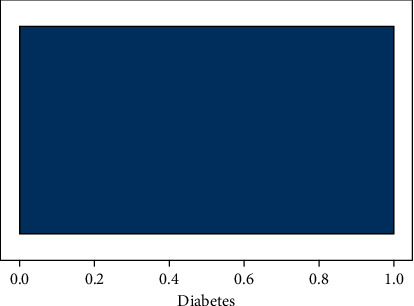
Diabetes induction rate per patient.

Following data balancing techniques were used in this research :

- Oversampling at randomly (imblearn): a technique for correcting datasets with inconsistency is called Random-Under Sampler. This method enables quick and simple information validation. Data are selected at random for each target group. Each target group's data are chosen at random. To evaluate the number class, select random samples with or without modification.
- Using a random oversample (imblearn): to handle the problem of partiality, develop a fresh minority sample size. The simplest method is to manually select new samples to remove the original ones.
- Under sampling (Tomek links): tomek linkages and opposing grouping pairs have several similarities. By expanding the zone between the two classes and removing each pair's higher class instances, the classification is made more accurate. Given that the two samples are close to one another Tomek's link is relevant.
- Using oversampling (SMOTE) :  the approach basis yields false data for the minority. SMOTE produces a random point from the minority community using SMOTE (synthetic minority oversample method). All folks of this place are also computed. Among the chosen point and its neighbours, the synthetic points in Figure 6 are added.

**Figure 7:**
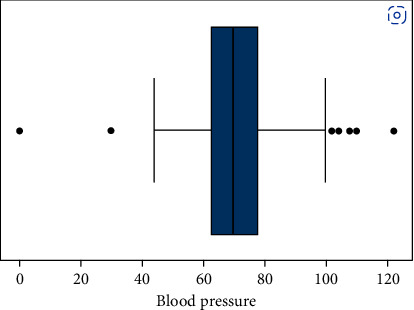
Blood pressure induction rate per patient.

**Figure 8:**
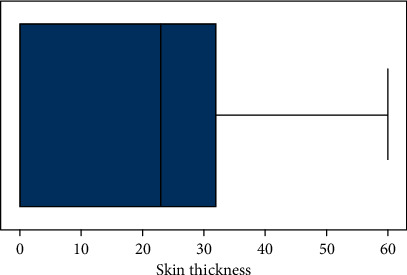
Skin thickness induction rate per patient.

**Figure 9:**
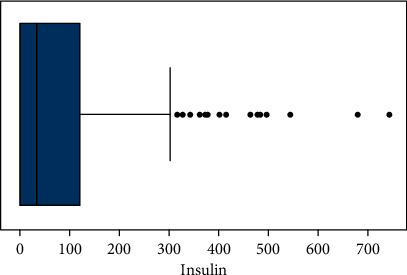
Insulin induction rate per patient.

When a boxplot data exceeds a certain range, the IQR technique is used to remove outliers. Determined by the interquartile range the variation between the top and bottom quartiles (IQR). Moreover, the interquartile range separates the top and bottom quartiles (IQR). Outliers in the research's data were found using statistical techniques like Inter - quartile, Z-Score, and dataset smoothing. The IQR is created by combining the first and third quartiles of a data collection, or the 25th and 75th decile, and is then produced by deducting Q1 from Q3, as depicted in Figures 6–9.

(1) $$\begin{array}{c} IQR=Q3-Q1\text{.} \end{array}$$

### 3.3. Feature Engineering

This method involved utilizing information from a specific space to foster capabilities that might be utilized by MLA. It is the extraction and transformation of raw data into MLA representations. The study uses a correlation matrix to identify the correlations between different data.

#### 3.3.1. Correlation Matrix

**Figure 10:**
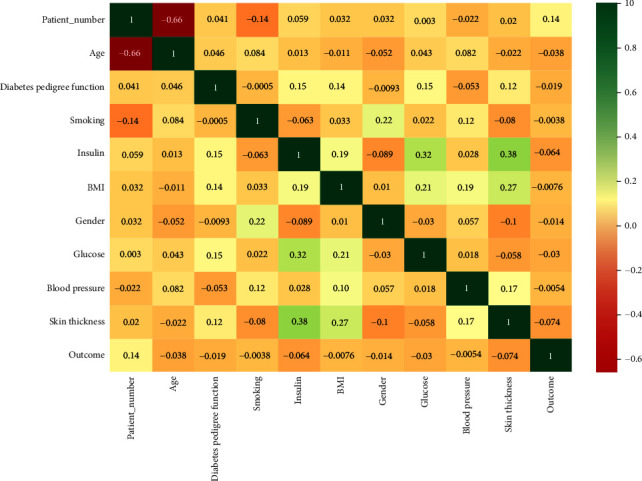
Correlation matrix of clinical dataset.

A correlation matrix is a covariance matrix. The concept of correlation describes the frequency and direction of a straight line link between two quantitative variables. Moreover, the correlation sums up the strength of the linear link. *R* represents the range of values between 1 and 1. Patient-Number and Age has no impact on either of these variables as shown by the negative correlation in Figure 10.

### 3.4. Cross Validation

Cross-validation in Machine Learning is the procedure of assessing methods with a small data sample. Moreover, *k* is the only parameter that controls how many groups of data should be generated from a specific collection, and controls the process. This technique is additionally known as K-fold cross-validation.

#### 3.4.1. K- Fold Cross Validation

Depending on the degree of data, divide the entire dataset into *K*-folds using a value among 5 and 10. Fit the model to folds *K*−1 and the remaining fold *K* to test it (*K* minus 1).

### 3.5. Classification of Algorithms

We outline an application of the MLA being researched for the early prediction of Diabetes in this section. Six effective classifiers are utilized to predict diabetes using the clinical dataset. Seven classifiers are applied for classification, namely, RF, MLP, SVC, DT, GBC, and LR algorithms. The voting classifier is evaluated using a combination of these six classifications that are the most accurate.

#### 3.5.1. Linear Regression (LR)

This method predicts the class of numerical variable. To predict binary results (*y* = 0 or 1) LR use statistical techniques. The possibilities of an event occurring are used to make LR predictions. The sigmoid function in the LR algorithm maps each data point. The standard logistic function results in an S-shaped curve. The sigmoid function is displayed in the following equation:

(2) $$\begin{array}{c} \text{Sigmoid equation}=1+\frac{1}{1+e^{-∆\left(-x\right)}}\text{.} \end{array}$$

#### 3.5.2. Decision Tree (DT)

A supervised MLA called Decision Tree (DT) was developed for classification. In principle, it is organised like a tree, with each inner (non-leaf) nodes denoting features each leaf node indicating a class prediction and each branch denoting the result of the test. The prediction model splits the data set repetitively based on a parameter that maximises data partition, generating a treelike structure. Information gain is the test that is most frequently used. According to the information gain, each split represents the highest level of entropy that is lost as a result of the split. The percentage of the values in the *y* class to all the features in the leaf node that includes the data item *x* is the estimate of *P* (*y* |*x*) [27].

#### 3.5.3. Gradient Boost Classifier (GBC)

Algorithms in groups termed as GBC these algorithms integrate several limited learning models to provide a reliable prediction. Using DT to raise the gradient is standard procedure. GBC is an MLA that creates a prediction model from a group of mediocre models can be used to address classification methods problems. GB in contrast to RF is decision trees with a weak learner and frequently outperforms them. As compared to applying the model sequentially, as other techniques would itself lessening an infinite differentiable loss function.

#### 3.5.4. Support Vector Machine (SVM)

SVM the hyperplane is constructed among various classes or objects. Calculating the size of the problem space produces the hyperplane. SVM also allows for data reduction to balance data dimensions. Using the support vectors and class corner points, the marginal distance between the classes is calculated from the centre of the hyperplane. Some of the variables used in SVM are kernels, C coefficients, and intercepts. The most crucial component of SVC is the kernel. Depending on the kind of data they receive, these kernels have been adjusted. The fact that the data are linear to RBF justifies the study's use of linear and Gaussian kernels [28].

#### 3.5.5. Random Forest (RF)

In the RF ensemble learning technique for classification as well as other tasks, decision trees is constructed uses the training and the best tree estimate. The majority vote method is employed at the classification stage to produce meaningful results in determining the kind of diabetic diseases. The outcomes of a healthy patient could be classified as either healthy or ill.

#### 3.5.6. Multilayer Perceptron (MLP)

A neural network's layers contain hidden, input, and output layers. The input layer accepts the data, while the output layer provides the results. Between the input layer and the output layer is a hidden layer. The body's neural network served as inspiration for the neural network. Similar to human neurons, network neurons exhibit probabilistic behaviour. The processing time in neural networks is significantly longer. In Weka, it is also known as a multilayer perceptron.

#### 3.5.7. Ensemble Learning

By combining lots of different classifiers into the system's classification accuracy can be improved. Whenever working on the same problem, two or more Machine Learning algorithms combine to improve the accuracy of classification.

#### 3.5.8. Performance Analysis

Recall, Precision, F- measure, MCC, and ROC Area are some of the performance measures to validate the techniques.

#### 3.5.9. Comparative Analysis of Existing Work

Comparisons have been made between the effectiveness of our suggested framework utilizing a variety of relevant forms of literature, methodologies, and analysis in data set. It was discovered that our thought-out framework yielded positive results for a variety of evaluation measures, especially accuracy for diabetes disease risk prediction. Various methods such as data Imputation is used to handle missing values and replace boxplot technique outliers as well as standard and balancing To attain better results data transformation techniques have been applied. Outcomes compared to similar works. Also, ensemble method and *k* Fold validation Technique while developing the proposed framework to achieve more valid results than other related studies.

## 4. Model Accuracy

**Figure 11:**
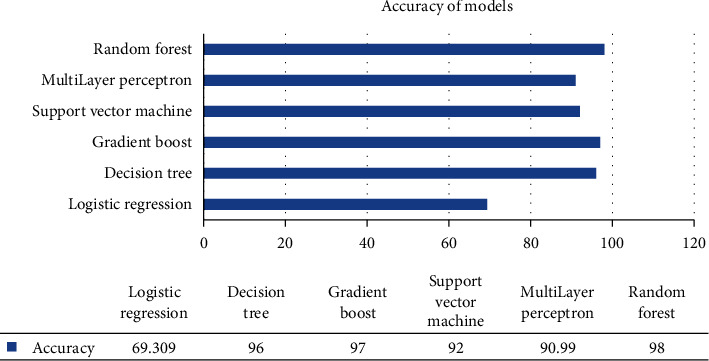
Bar graph for accuracy of model.

We can better understand the changes by displaying the accuracy values in Figure 11 which shows a comparison of multiple. MLA based on the accuracy amongst them. The result compares that RF has the highest accuracy than the other models. Bar graph shows the accuracy of various algorithms depicted as follows.

## 5. Result and Discussion

**Figure 12:**
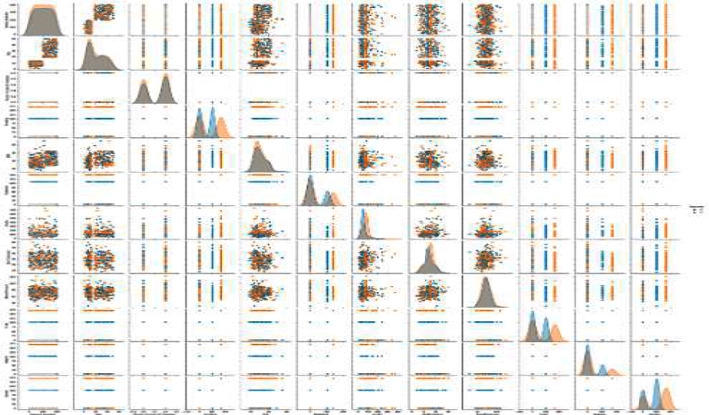
Depiction of scatterplot feature of data.

Six algorithms were applied such as RF, MLP, SVC, DT, GBC, and LR algorithms. The top-performing algorithm was Random Forest. We select the top-performing algorithms from each portion and closely reviewed the outcomes. The splitting outcomes of MLA can produce accurate, reliable, and tested in some cases they may even outperform learnable ability. The scatterplot in Figure 12 depicts the relationships between the features that have been utilized to create the dataset. Every dot position values used to quantify each data point are shown along the *X* and *Y* axes.

**Figure 13:**
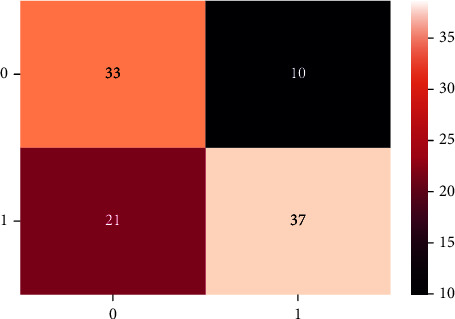
LR algorithm.

**Figure 14:**
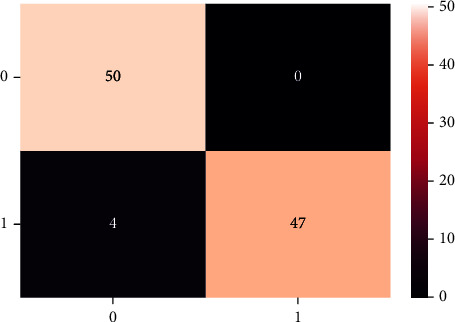
DT algorithm.

**Figure 15:**
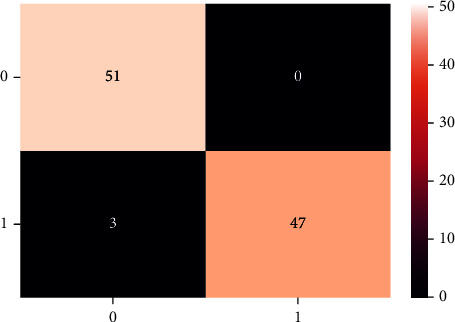
GBC algorithm.

**Figure 16:**
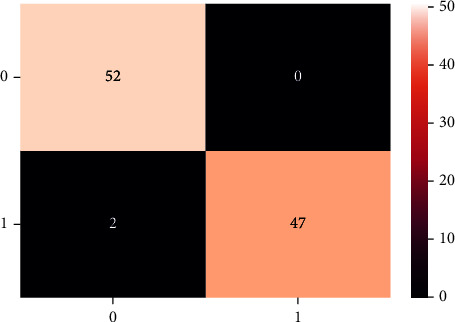
RF algorithm.

**Figure 17:**
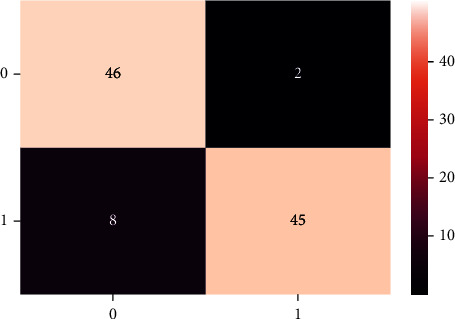
MLP algorithm.

**Figure 18:**
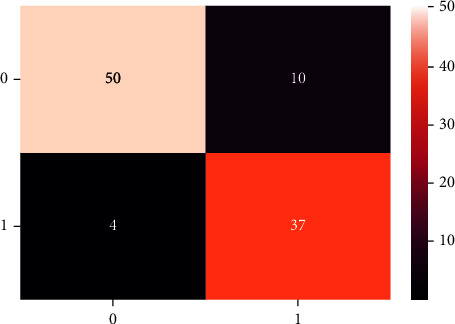
SVM algorithm.

**Figure 19:**
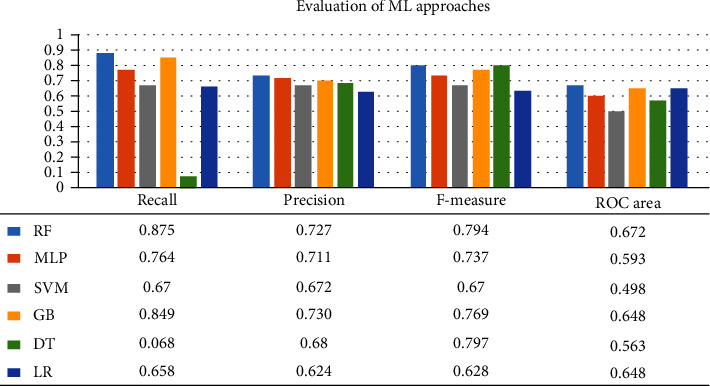
Evaluation of the effectiveness of ML approaches.

A confusion matrix is used in the Machine Learning model to evaluate how well the algorithms perform. The confusion matrices have been utilized to assess the various MLT through statistical metrics like precision, recall, specificity, F- measure, MCC, and ROC Area. It has a tabular layout where the rows are the actual values and the columns are the predicted values. In Figures 13–18 these classifiers confusion matrices are displayed. The Clinical diabetes dataset utilized in this study is shown in Table 2 in order to better diagnose and predict early diabetes using MLA. In addition, as depicted in Figure 19 various further statistical measures are also calculated. The Machine Learning models are validated using these variables.

Table 2 compares the Clinical dataset utilized in this study with the PIMA diabetes dataset in order to better diagnose Early Prediction of Diabetes based on the Machine Learning Algorithm as compared to the diabetes clinical data set has more accuracy.

## 6. Conclusion and Future Scope

Diabetes is a serious and chronic condition. Diabetes can be detected early enough which can result in more effective treatment. This study also compares various classification models based on machine learning algorithms for predicting a patient's diabetic condition at the earliest feasible stage. After dataset balancing, classifiers' accuracy was compared. The prime objective of our research is to determine the early prediction of diabetes using the state of advanced MLA in one of the rural areas of North Kashmir. The data set employed for this experimentation was gathered from clinical professional. In the medical diagnosis, we used diabetes clinical data set with 403 instances and 11 attributes. The professionals (Prediabetes specialists) in the medical field have approved the features chosen for the early diagnosis of diabetes Prediction. The prevalence of diabetes is showing an upward trend in Bandipora Kashmir. It is recommended that using state of art algorithms for the early prediction can help in decreasing the upward trend of diabetes Six algorithms including RF, MLP, SVM, DT, GBC, and LR algorithms were utilized for this purpose amongst all algorithms we achieved RF has the highest accuracy of 98%. RF also has produced successful outcomes for several statistical metrics includes ROC Area, Recall, Precision, F-measure, and MCC. K-fold Machine Learning models such as cross-validation has been used to evaluate RF, MLP, SVM, DT, GBC, and LR. The framework utilized in this research will be applied to ensemble and hybridization Machine Learning in order to further recent research. In the future, a more comparison analysis between various datasets and their features can be conducted in order to identify all the crucial features for forecasting diabetes. To determine the best and most accurate diabetes prediction algorithm, a variety of various algorithms and combinations of algorithms can be examined.

## Figures and Tables

**Table 1:** Comparative Study on performance metrics.

S. No.	Ref no/Paper published	Algorithm/Tech used	Database set used	Results
1	Reference [18]/IEEE	KNN, NB, DT	PIMA dataset	KNN74.89%, NB 92.6%, DT76.9%
2	Reference [19]/Elsevier	SVM, NB, J48	PIMA dataset	SVM83.11%, NB, 86.53 percent for sensitivity
				J4876 percent accuracy
3	Reference [20]/Springer	K-mean, LR, SVM, Gaussian model	PIMA dataset	K-mean 95.42%
4	Reference [10]/Springer	NB, RF	PIMA dataset	NB87.94%, RF67%
5	Reference [21]/Hindawi	KNN, SVM	PIMA dataset	92.82 percent means selected 511 samples from the 768 cases, and 257 samples were identified as outliers.
6	Reference [22]/Wiley	Bayesian approach to classification	PIMA dataset	Greater than 87% accuracy
7	Reference [23]/IEEE	SVM, KNN	PIMA dataset	SVM 78%, KNN79%
8	Reference [24]/Scopus	RF, DT, MLP	PIMA dataset	RF80%,DT77%, MLP76%
9	Reference [25]/Springer	SVM, KNN	Type 2 diabetes dataset	SVM80%, KNN 76%
10	Reference [26]/Nature	RF, NB, DT	PIMA dataset	RF76%, NB75%, DT75%.

**Table 2:** Accuracy of MLA model.

Classifiers	Accuracy with PIMA data set	Accuracy with the clinical data set
Logistic regression	62 [42]	69.309
Decision tree	73.82 [43]	96
Gradient boost	87 [44]	97
Support vector machine	73 [45]	92
Random forest	92 [46]	98
Multilayer perceptron	83 [47]	90.99

## Data Availability

The processed data are available upon request from the corresponding author.
